# Feasibility and Safety of Very-Low Contrast Combined Ringer's Solution in Optical Coherence Tomography Imaging

**DOI:** 10.3389/fcvm.2022.844114

**Published:** 2022-03-24

**Authors:** Tao Chen, Huai Yu, Lijia Ma, Chao Fang, Haibo Jia, Huimin Liu, Maoen Xu, Donghui Zhang, Guang Yang, Shuangyin Zhang, Jincheng Han, Guo Wei, Yanchao Liu, Jingbo Hou, Bo Yu

**Affiliations:** ^1^Department of Cardiology, The Second Affiliated Hospital of Harbin Medical University, Harbin, China; ^2^The Key Laboratory of Myocardial Ischemia, Chinese Ministry of Education, Harbin, China

**Keywords:** optical coherence tomography, acute coronary syndrome, contrast media, Ringer's solution, atherosclerosis

## Abstract

**Background:**

Optical coherence tomography (OCT) is an important modality used in coronary intervention. However, OCT requires a high amount of contrast media, limiting its extensive application in clinical practice. This study compared OCT images of coronary lesions obtained using contrast media and very-low contrast combined Ringer's solution (VLCCR) in patients with acute coronary syndrome (ACS).

**Methods:**

Thirty ACS patients with a total of 36 native lesions and stenoses from 70 to 90% were included in this study. Two kinds of flushing media (a contrast medium and VLCCR) were used in succession in a random order for OCT image pullback of each lesion. VLCCR method is using low volume contrast (4–5 ml) injected into the guiding catheter previously combination with injector infused Ringer's solution instead of pure contrast medium. The safety of procedure was evaluated by recording the patients ‘symptoms, changes of ECG, blood pressure and heart rate. OCT images were analyzed to determine the image clarity. Lumen area and diameter were also measured and the consistency between the two media was compared.

**Results:**

OCT procedure using either contrast or VLCCR did not show any peri-procedural adverse events. There was no difference in changes of blood pressure and heart rate in both procedures, however, VLCCR procedure showed less procedure-related symptoms and ECG changes. We found that the percentage of clear image frame was equivalent between the contrast and VLCCR media (98.0 vs. 96.9%, *P* = 0.90). We also observed a high degree of similarity between the different lesion phenotypes of ACS for both media. There was a linear correlation of the phenotypes obtained with these two different methods, and a significant correlation was observed between measurements obtained with contrast and VLCCR without correction for the refractive index of VLCCR (correlation coefficients ranged between 0.829 and 0.948).

**Conclusions:**

OCT imaging using VLCCR for blood clearance is feasible and safe and provides similar imaging quality compared to OCT imaging obtained using radiographic contrast media for ACS patients.

## Introduction

Intravascular optical coherence tomography (OCT) is a high-resolution imaging modality used to evaluate microstructural characteristics of coronary plaques and other findings during and post percutaneous coronary intervention (PCI) procedure (1, 2). Due to technological advances in the past several years, the utilization of OCT has been increasing, during which injection of contrast media is used to replace the occlusion technique. The current generation of OCT systems allows acquisition of images of a target vessel in a few seconds with nearly instantaneous display of the longitudinal lumen contour with a high pullback speed. However, OCT technology has some limitations. For instance, it has signal attenuation by red blood cells (RBCs) and requires continuous flushing with a medium usually used for radiographic contrast for low penetration depth and image acquisition. In addition, previous studies have shown that OCT image acquisition requires a high amount of contrast media, which can potentially cause side/toxic effects including renal dysfunction, cardiotoxicity, and seizures (3–5).

Several media are being used in clinical practice as flushing media for OCT image acquisition, with each having advantages and disadvantages. For instance, perfluorodecalin (PFD) has high viscosity, displaces blood, and generates clear OCT images, but has not been approved by the Food and Drug Administration and thus cannot be applied in the clinic due to its toxicity (6). Iohexol is a common contrast medium used for OCT flushing with lower toxicity than iodized contrast agents (7). Dextran decreases scattering of RBCs by matching refractive indexes between blood cells and blood plasma. Several studies have shown that low-molecular-weight dextran (LMWD), which exhibits minimal toxicity compared to contrast agents, could be used as a flushing medium to acquire OCT images (8–10). Recently, studies also have evaluated the feasibility of heparinized saline as flushing media for OCT image acquisition during PCI optimization (11, 12).

Ringer's solution is a sodium chloride solution that can be used instead of normal saline to adjust body fluid, electrolyte, and acid-base balance (13). However, Ringer's solution cannot be used alone as a flushing medium to acquire clear OCT images because of its low viscosity cannot flush blood clearly in short time. Therefore, it will be an excellent method to acquire perfect OCT imaging if only used low volume contrast combined with Ringer's solution.

In the present study, we compared quality and quantitative measurements of OCT images between very-low contrast combined Ringer's solution (VLCCR) and regular contrast medium to assess culprit lesions of patients with acute coronary syndrome (ACS).

## Methods

### Study Population

This single-center, prospective, and observational study recruited 30 ACS patients who were hospitalized at The Second Affiliated Hospital of Harbin Medical University. Patients with any one of the following were excluded from the study: ST-segment elevation myocardial infarction (STEMI) within 24 h, New York Heart Association class III or IV heart failure, unstable hemodynamic status, severe coronary tortuosity, severe coronary calcification, or one of the following lesions: (1) vessel diameter <2.5 mm or >5.0 mm, (2) total occlusion or obvious thrombosis, (3) lesion in a highly tortuous vessel, (4) ostial lesion, or (5) a Thrombolysis in Myocardial Infarction (TIMI) flow grade ≤2.

STEMI was defined as intense and unremitting chest pain for >30 min with patient's arrival at the hospital within 12 h from symptom onset, a new left bundle-branch block on the 12-lead electrocardiogram (ECG) or ST-segment elevation >0.1 mV on more than two contiguous leads, and elevated levels of serum cardiac markers (creatine kinase-MB or troponin T/I). NSTE-ACS included unstable angina pectoris (UAP) and non-ST segment elevation myocardial infarction (NSTEMI). NSTEMI was defined as elevated levels of serum cardiac markers accompanied by ischemic symptoms without ST elevation on the ECG. UAP was defined as rest angina or newly developed/accelerating chest pain on exertion within 2 weeks. The presence of a culprit lesion was identified using s stress test, coronary angiogram, ECG, or echocardiogram.

The study protocol was approved by the Institutional Ethics Committee of Harbin Medical University. The approval number was 2016YFC1301103. Written informed consent was obtained from all patients prior to the initiation of this study.

### Angiography

Coronary angiography was carried out through radial or femoral access over a conventional guide-wire after injection of 200 μg nitroglycerin. Images of all lesions were captured from at least two orthogonal views. The physicians decided to select anticoagulation, other adjunctive pharmacotherapy, and PCI technique and equipment for individual patients as appropriate. Qualitative coronary angiographic (QCA) analyses were performed to evaluate lesion location and complexity and TIMI flow grade and measure variables, including minimal lumen diameter (MLD), lesion length, % diameter stenosis, reference vessel diameter, and TIMI flow grade.

### OCT and Image Acquisition

OCT was carried out in all patients using a 6-Fr guiding catheter through the femoral, radial, or brachial artery after angioplasty or angiogram. The EBU (Medtronic, USA) and XBRCA (Johnson & Johnson, Cordis, USA) guiding catheters were used in the left and right coronary arteries. An intracoronary injection of 1.0–2.0 mg nitroglycerin and intravenous (i.v.) bolus injection of 100 IU/kg heparin were carried out for all patients before the OCT procedure.

All OCT images were acquired using the Dragonfly OPTIS Imaging Catheter (Abbott Vascular) and the intravascular catheter system (ILUMIEN OCT Imaging system; Abbott Vascular, Santa Clara, CA, USA). In each vessel, OCT was performed with two different continuous flushing methods in succession in a random order for each OCT image pullback. The conventional OCT procedure was carried out using manual injection of the regular contrast medium iodixanol 320 mg I/ml (GE Healthcare, Princeton, NJ, USA) as previously described (14). In this study, each pullback of OCT using conventional procedure needs about 11–12 ml contrast by manual injection. The novel OCT procedure with VLCCR involved three steps: (i) the OCT catheter was placed to cover the target lesion; (ii) the low volume contrast was injected into the guiding catheter to ensure that it had goodcoaxial position; and (iii) sodium lactate Ringer's solution (Qidu, Shandong, China) was infused through an automated injector (MEDRAD *Mark 7* Arterion, Bayer, Germany, parameter settings: 6.0 ml/s, 300 psi) into the coronary artery. VLCCR used in left coronary system (LAD, LCX or Diagonal branch) contained 4.7 ml contrast, while it used in RCA contained 4.1 ml contrast. Each pullback of OCT using VLCCR needs about 8–9 ml Ringer's solution by automated injector. Automatic OCT pull-back was taken for length of 75 mm at the speed of 36 mm/s using both flushing media. This mode was taken because of its easy to clear blood for short time using low viscosity flushing medium. All procedures were performed by well-trained operators.

### Safety Assessment

During the injection of each flushing medium for image acquisition, peri-procedural adverse events (abrupt vessel occlusion, hematoma, hemorrhage, vessel thrombosis), ECG changes, beat-by-beat hemodynamics, and uncomfortable symptoms (if any) were recorded. The same coronary vessel segment was sequentially imaged during intracoronary injection of Ringer's solution and radiographic contrast at room temperature.

### OCT Analysis

Two independent experienced observers (T.C and H.Y), who were blinded to the type of flush medium, analyzed all OCT frames in the pullbacks from the OCT system using offline software (Abbott Vascular). Each OCT pullback was examined by observers on a frame-by-frame basis, and any discrepancies between the two observers were resolved through a consensus form a third investigator (H.J.). The segments analyzed were culprit lesions centered on the narrowest position, 5 mm to the distal and proximal ends.

**Figure 1:**
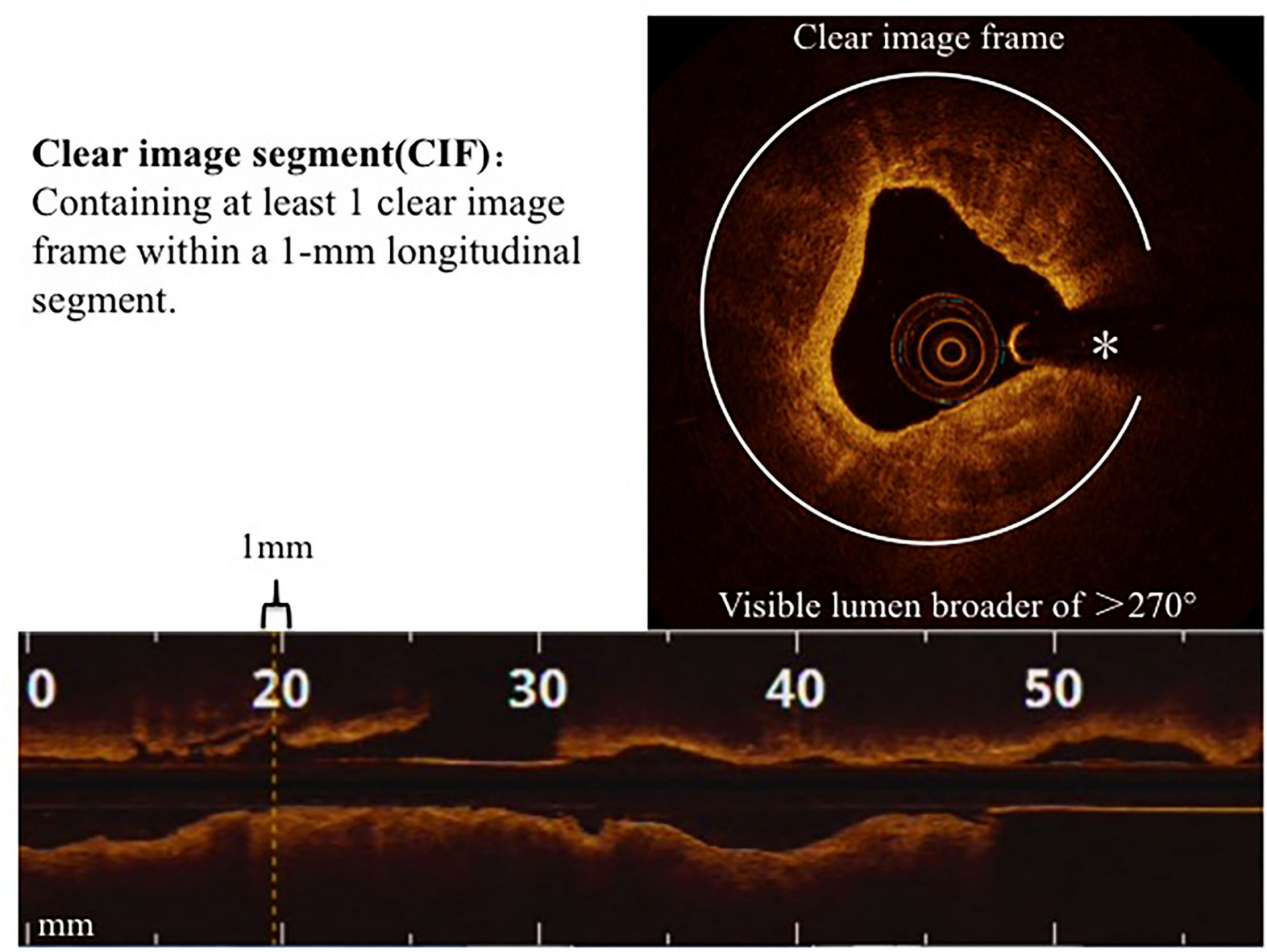
Clear image segments (CIS). CIS and CIF are defined in the Methods section. *Guide-wire artifact.

The cumulative number of clear image segments (CIS) obtained from the entire image of a culprit lesion, which was defined as those harboring at least 1 clear image frame (CIF) within a 1-mm longitudinal segment (Figure 1), was the comparative parameter. The length of the analyzed segment was designated as 5 mm that covered the most severe stenosis of the culprit lesion. CIF was designated as an OCT cross-sectional image frame where the boundary between the vessel wall and the lumen was distinguishable along a continuous arc of at least 270° in relation to the lumen center as previously detailed (15). The following parameters, maximum lumen diameter (MaxLD), minimum lumen diameter (MinLD), mean lumen diameter (Mean LD), and minimum lumen area (MLA), were measured at every 1-mm on the cross section. Lesion morphology was classified as normal, fibrotic, calcified, or lipid-laden, as previously described (4).

### Statistical Analysis

The normality of continuous data was examined using the Kolmogorov–Smirnov test. Continuous variables are presented as mean ± standard deviation (SD) for normally distributed variables and as median (25th−75th percentiles) for non-normally distributed data. Data were statistically compared between groups using an independent sample *t*-test or the Mann–Whitney *U-*test, as appropriate. Categorical data are presented as counts (proportions) and were statistically compared between groups using the χ^2^ test or Fisher's exact test (if the expected cell value was <5). The correlation between these two methods was analyzed using Pearson's correlation coefficients. A two-tailed *P*-value <0.05 was considered statistically significant. All statistical analyses were carried out using SPSS version 19.0 (SPSS IBM, Armonk, New York, USA).

## Results

### Demographic and Baseline Clinical Characteristics of Patients

Table 1 lists demographic and baseline clinical characteristics of patients at the time of PCI procedure; a total of 30 patients with 36 vessel OCT images were included in this study. The following vessels were imaged: right coronary artery (5/36, 13.9%), left anterior descending artery (26/36, 72.2%), circumflex or obtuse marginal artery (4/36, 11.1%), and diagonal artery (1/36, 2.8%) (Table 2).

**Table 1:** Baseline characteristics of the patient population.

**Variable**	**All patients (*n* = 30)**
Age (years)	61 (40–79)
≥65 years, *n* (%)	11 (36.7)
Sex (F/M), *n* (%)	8/22 (26.7,73.3)
Hypertension, *n* (%)	13 (43.3)
Diabetes mellitus, *n* (%)	8 (26.7)
Current smoker, *n* (%)	15 (50.0)
TnI (μg/L)	25.35 (0–377.816)
CK-MB (μg/L)	32.05 (0–338.2)
WBC (× 10^9^/L)	8.21 (4.5–13.8)
CRP (mg/L)	5.21 (0.22–15.26)
Glucose (mmol/L)	7.23 (3.89–25.59)
TC (mmol/L)	4.27 (2.1–6.37)
TG (mmol/L)	1.68 (0.61–5.65)
HDL-C (mmol/L)	0.99 (0.61–1.6)
LDL-C (mmol/L)	2.7 (0.95–4.78)
HbA1c (%)	6.34 (4.8–10.8)
BUN (mmol/L)	6.85 (3.52–18.16)
Ccr (μmol/L)	86 (49-338)
BNP (pg/mL)	665 (18–4976)
**Medical therapy**	
Anti-platelet, *n* (%)	30 (100)
β-bloker, *n* (%)	15 (50)
ACEI or ARB, *n* (%)	7 (23.3)
CCB, *n* (%)	8 (26.7)
Insulin, *n* (%)	5 (16.7)
Statin, *n* (%)	28 (93.3)

*Values expressed as n (%), mean ± SD or median (IQR). ACEI, angiotensin-converting enzyme inhibitor; ARB, angiotensin receptor blocker; BNP, brain natriuretic peptide; BUN, blood urea nitrogen; CCB, calcium channel blocker; Ccr, creatinine clearance rate; CRP, C-reactive protein; TC, total cholesterol; TG, triglyceride; HDL-C, high-density lipoprotein cholesterol; LDL-C, low-density lipoprotein cholesterol; WBC, white blood cell*.

**Table 2:** Angiographic characteristics of the culprit vessel.

**Variable**	**All vessels (*n* = 36)**
**Culprit vessels**, ***n*** **(%)**	
LAD	24 (72.2%)
LCX	4 (11.1%)
RCA	5 (13.9%)
D1	1 (2.8%)
Minimal lumen diameter (mm)	0.88 (0.13–1.84)
Reference vessel diameter (mm)	2.86 (2.12–4.28)
Diameter stenosis (%)	69.31 (29–95)
Lesion length (mm)	19.34 (6.88–42.68)

*Values expressed as n (%), mean ± SD or median (IQR). LAD, left anterior descending artery; LCX, left circumflex artery; RCA, right coronary artery; D1, diagnose artery 1*.

### Safety and OCT Catheter Performance

**Figure 2:**
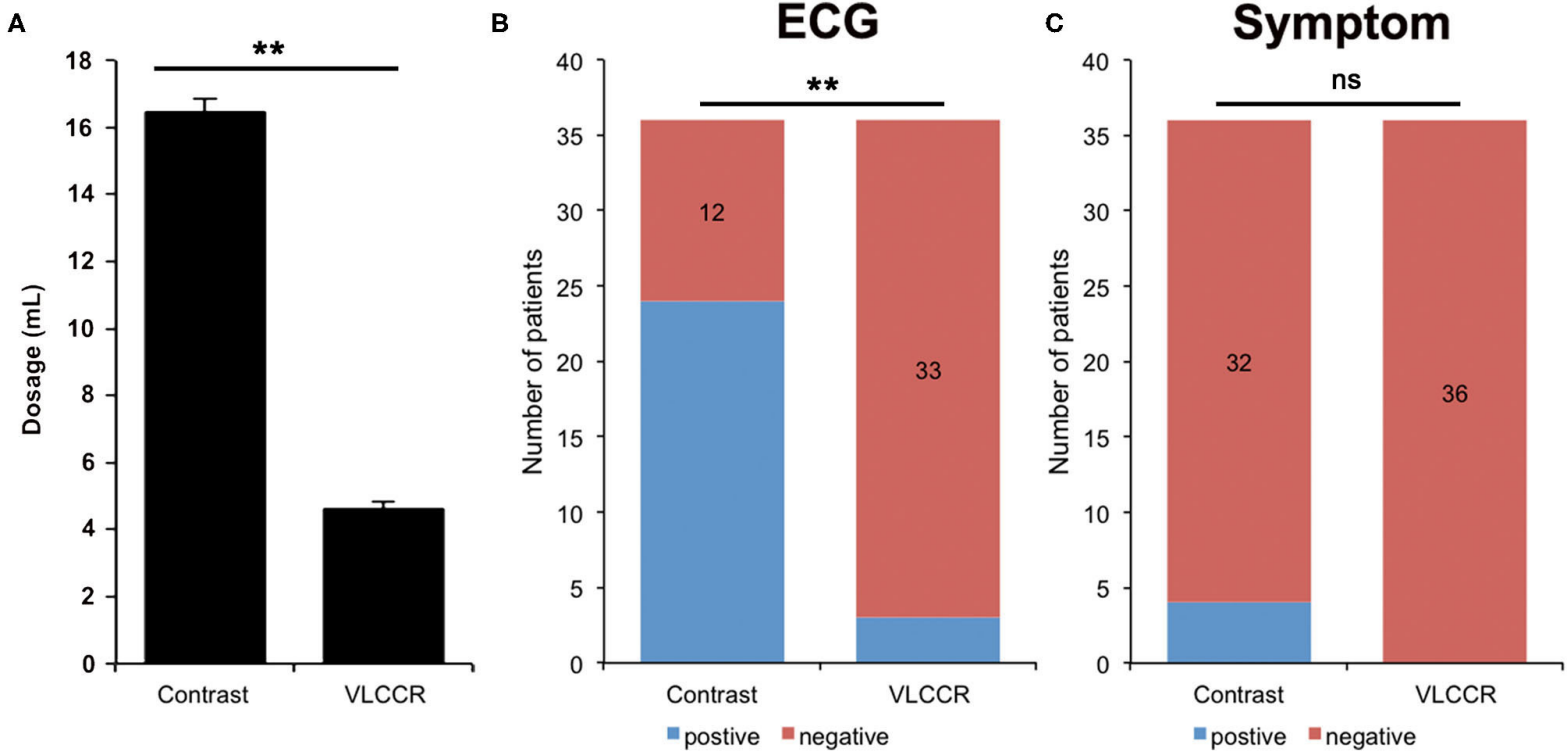
Comparison of the amount of contrast medium and VLCCR used **(A)**, ECG **(B)**, and symptoms **(C)** of ACS patients (*n* = 30) **P < 0.01. ns, no significant difference.

All 30 patients received an intravascular OCT examination and did not show any peri-procedural adverse events, such as abrupt vessel occlusion, hematoma, hemorrhage, vessel thrombosis, or significant hemodynamic changes. The regular contrast medium had a significantly higher volume injected for imaging than VLCCR (Figure 2A). No clinically relevant fluctuations or deviations were observed during intraoperative monitoring of blood pressure and heart rate before each administration of the flush medium. The mean systolic blood pressure and heart rate for all runs were 145.2 ± 21.5 mmHg and 70.5 ±10.3 beats per minutes (bpm), respectively. During the contrast flushing OCT imaging, more procedure-related ECG changes, including ST-segment depression, bradyarrhythmia, bundle branch block, and chest oppression or pain, occurred (Figures 2B,C).

### Evaluation of Image Quality

We next evaluated the total length of segments of 360 mm for both the contrast medium and VLCCR groups. The number of CIS was 353 (98.0%) in the contrast medium group and 349 (96.9%) in the VLCCR group (*P* = 0.90). A total of 3,600 cross-sections (1,800 per group) were analyzed. Nearly all cross-sections were clearly imaged in both groups except those segments where blood was not flushed off by the contrast medium or VLCCR due to severe stenosis.

### Plaque Characterization

**Figure 3:**
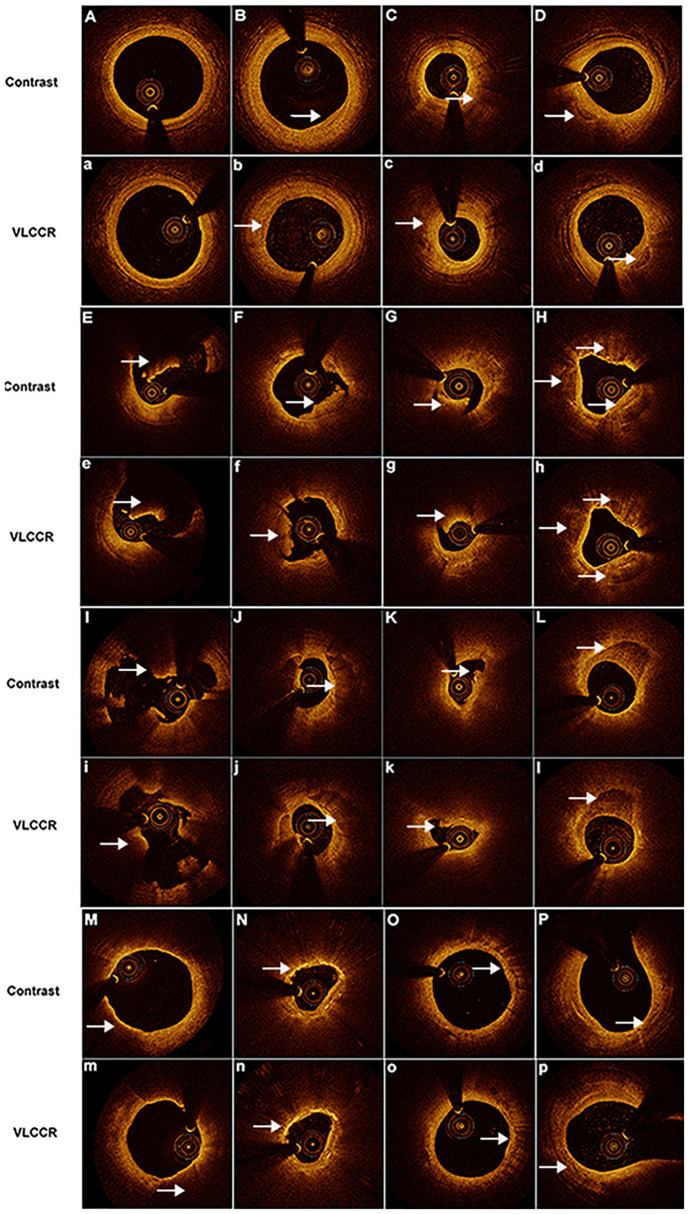
Representative images obtained by OCT with contrast media and VLCCR at the culprit lesion site in ACS patients with different coronary atherosclerotic plaques. Clear images were acquired with both contrast media (Capital) and VLCCR (lower case). **(A,a)** normal vessel; **(B,b)** fibrous plaque; **(C,c)** lipid-laden plaque; **(D,d)** fibrocalcific plaque; **(E,e)** red thrombus; **(F,f)** white thrombus; **(G,g)** mixed thrombus; **(H,h)** healed plaque; **(I,i)** plaque rupture; **(J,j, K,k)** plaque erosion; **(L,l)** calcific plaque; **(M,n)** thin-cap fibroatheroma; **(N,n)** cholesterol crystal; **(O,o)** macrophage; and **(P,p)** microchannel. White arrow indicates the imaging characteristic of plaques.

Plaque morphology, including fibrous, lipid-laden, calcium, rupture, erosive, healed, and thrombus plaques, of all target lesions in ACS patients was characterized and compared between the contrast medium and VLCCR groups. The contrast medium and VLCCR groups had comparable plaque classifications (Figure 3).

### Quantitative Assessment

**Figure 4:**
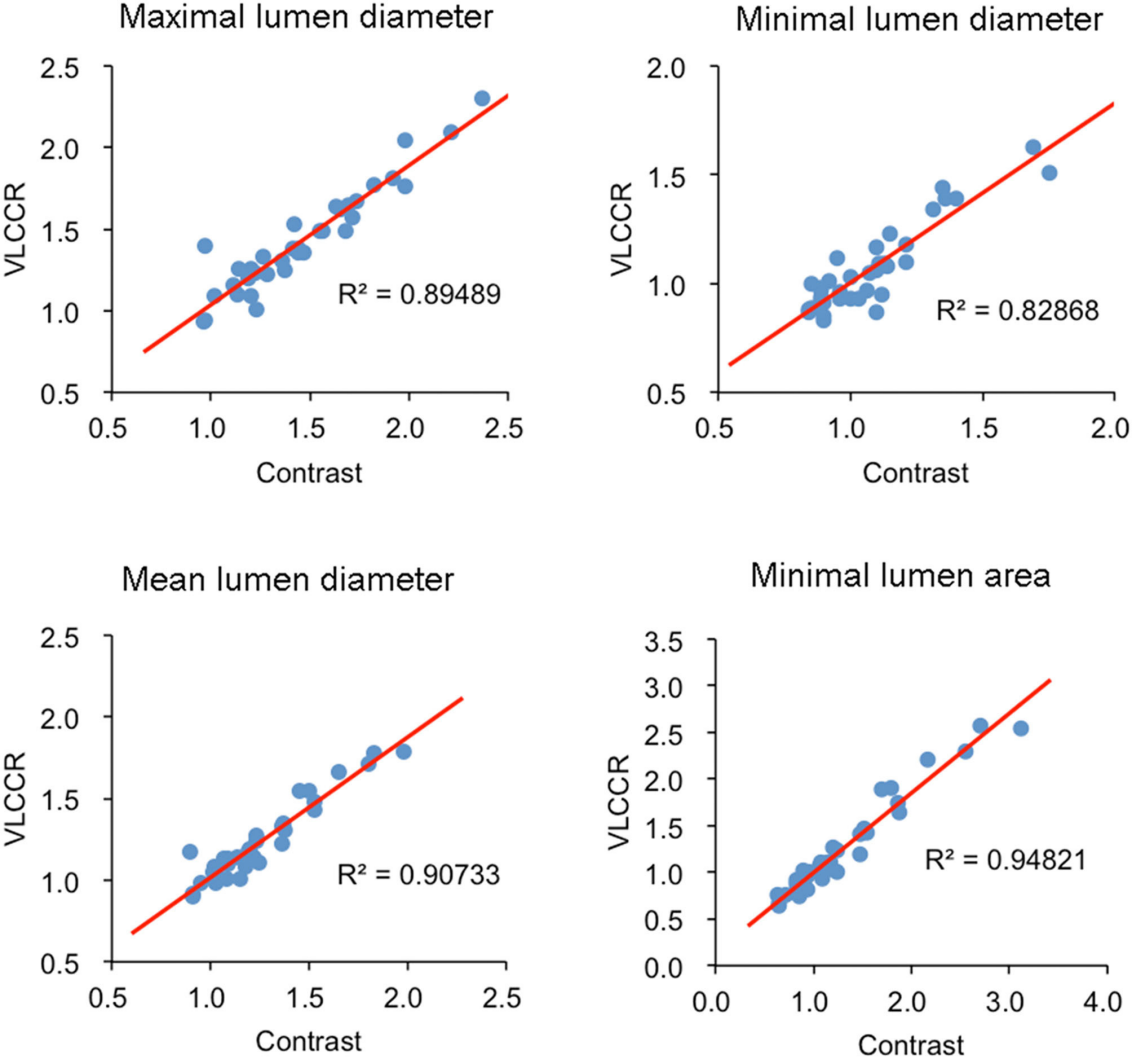
Correlations of MaxLD, MinLD, Mean LD, and MLA between contrast medium and VLCCR groups.

Linear regression analysis was performed to examine the correlations of MaxLD, MinLD, Mean LD, and MLA in two pullbacks between the contrast medium and VLCCR groups (Table 3). Both groups had high correlation coefficients for all parameters with no significant differences (all *P* > 0.05). A linear correlation was observed between the phenotypes between the two different methods. A significant correlation was observed between measurements obtained with the contrast medium and VLCCR (correlation coefficients ranged between 0.829 and 0.948) (Figure 4).

**Table 3:** Comparison of OCT imaging performed using contrast vs. VLCCR.

**Variables**	**Contrast (*n* = 36)**	**VLCCR (*n* = 36)**	***P*-value**	**Correlation coefficient**
Clearly imaged cross-sections (%)	98.0%	96.9%	0.900	
Mean lumen area (mm^2^)	1.3 ± 0.6	1.3 ± 0.5	0.718	0.948
Mean lumen diameter (mm)	1.3 ± 0.3	1.2 ± 0.2	0.754	0.907
Minimal lumen diameter (mm)	1.1 ± 0.2	1.1 ± 0.2	0.855	0.829
Maximal lumen diameter (mm)	1.5 ± 0.4	1.4 ± 0.3	0.716	0.895

*Values expressed as n (%) or mean ± SD*.

*A p-value < 0.05 was considered statistically significant*.

*VLCCR, Very Low Contrast Combined Ringer's solution*.

## Discussion

In the present study, we compared the quality and qualitative measurements of OCT images acquired with contrast medium and VLCCR, and found that VLCCR and contrast medium groups had comparable image quality and lumen measurement of OCT imaging. Additionally, good correlations between the contrast medium and VLCCR groups were observed in the measurements of MaxLD, MinLD, mean LD, and MLA. However, the VLCCR group used a lower amount of contrast medium than the contrast medium group. Thus, we believe that VLCCR could potentially replace the contrast media for OCT image acquisition. To the best of our knowledge, this is the first study demonstrating the feasibility and safety of using VLCCR in OCT-guided PCI in clinical practice.

The incidence of high mortality and morbidity in ACS patients is affected by many confounding factors, such as age, diabetes, chronic kidney disease, left ventricular dysfunction, severity of coronary disease, and clinical manifestations of ACS, such as fatal arrhythmias, cardiogenic shock, and cardiac arrest. In particular, STEMI patients were reported to have nearly double the rates of contrast-induced acute kidney injury compared to other patients, which might be attributed to neuro-hormonal activation, inadequate opportunity for volume expansion, cardiac release of catalytic iron, and high incidence of hemodynamic instability (16, 17). Compared with intravascular ultrasound (IVUS), OCT technology has advanced with better technical performance and higher image quality, as well as convenient procedures such as automatic lumen measurements, faster pullback, and co-registration with angiography in the past decades (18). OCT also can provide more precise morphological information of native coronary artery lesions than other conventional intracoronary imaging modalities (14). In addition, accumulating evidence has shown that OCT may provide an individualized precision treatment strategy, which is a promising modality during primary PCI (19). Moreover, OCT-guided PCI was non-inferior to IVUS-guided PCI in terms of acute and long-term clinical outcomes (20). As a result, the recent European guideline recommended OCT for stent optimization as Class IIa (21). OCT is therefore expected to be more widely used in the clinic in the future. However, OCT requires flushing blood cells with contrast medium prior to intracoronary image acquisition, and also requires 17–70 mL more contrast medium than IVUS or angiographic guidance (22–24). Hence, OCT is potentially linked to a higher risk of complications related to the use of more contrast media. It is highly desirable to obtain high quality OCT images while reducing the amount of contrast media during primary PCI for complex lesions.

X-ray contrast media, such as iodixanol and iohexol, are frequently used to clear blood for OCT image acquisition (25, 26). However, the use of contrast media in some patients may cause dysfunction of many organs, including the most common organ damage, contrast-induced nephropathy (CIN). CIN is closely correlated with increased in-hospital stay and long-term morbidity and mortality (27), limiting the use of intravascular OCT. Thus, it is imperative to reduce the amount of contrast medium needed to clear blood to maximally diminish potential side effects.

LMWD has been used as the flushing medium for coronary angioscopy, and it was reported that OCT image acquisition with LMWD achieved the same image quality and quantity compared with OCT using contrast medium (10), Although LMWD itself did not shown any significant nephrotoxicity, LMWD might affect fluid reabsorption in the renal tubules during hypovolemia (28). Therefore, it is necessary to monitor the hypovolemic status when LMWD is used for OCT. Potential side effects caused by LMWD include anaphylactic reactions, coagulopathy, renal dysfunction, and volume overload. Furthermore, in recent studies the investigators also demonstrated the effectiveness of using heparinized saline as a flushing medium for OCT imaging to optimize the PCI procedure. This method does greatly reduce the volume of contrast agents used during PCI. SOCT PCI study was the first study to explore the feasibility, safety and efficacy of heparinized saline as flushing media for coronary OCT with the success rate of 88.1% (Effective runs for PCI that contains 61 good runs and 27.1% clinically usable runs) (12). Another study did head-to-head comparison of saline and contrast for image quality and measured vessel parameters and found it comparable (11). But these studies had limitations of small sample size, manual injections for both contrast as well as saline and single center studies. In the present study, the proportion of CIS obtained by VLCCR was 96.9%, while the proportion of CIS obtained by traditional method using pure contrast agent was 98.0%, and there was no statistical difference between them. VLCCR not only significantly decreased the total volume of contrast medium needed during OCT, but also did not reduce the quality and analyzability of the images. Thus, OCT with VLCCR as a flushing medium may benefit ACS patients, especially those with renal dysfunction.

Our study has important clinical potential: administration of radiographic contrast medium for blood clearance currently presents an important limitation for OCT imaging, because contrast medium administration is potentially linked to complications such as allergic reactions and CIN. Additionally, radiographic contrast medium is expensive, thus increasing the patient's financial burden. Although previous study has confirmed that the OCT imaging quality of pure contrast (iodixanol 320) was better than the other two methods including iodixanol 320 and Ringer's, iodixanol 320, and 50% albumin (27). However, this imaging method will use a large dose of contrast, and our improved version of the new method can ensure clear image quality while only using a low dose of contrast.

Therefore, OCT imaging with VLCCR is safer and less costly, which may be particularly beneficial to patients with baseline renal insufficiency or allergy. Because Ringer's solution does not have any significant side effects in clinical applications, the VLCCR application should be considered as a safe and effective way to replace the conventional contrast media during OCT imaging in the future.

The present work demonstrated that both OCT image acquisition methods, VLCCR and conventional contrast medium, obtained equivalent high-definition quality images and quantitative image measurements. However, measurements obtained with contrast medium and VLCCR were not completely correlated. This could be because the VLCCR method used a mix of a low volume of contrast medium with the Ringer's solution. The current OCT instrument design is mainly based on the value of the corresponding quantitative parameters calculated after the refractive index of the reference contrast medium. One of these OCT machines has been designed to contain function for transferring to the refractive index of the reference LMWD (VIVOLIGHT, China). Because the refractive indexes of the contrast medium and VLCCR were different during the imaging process, there is a need to design OCT intelligent software in the future that can accurately analyze a variety of blood flushing agents. This will aid in formulating appropriate flushing contrast media while retaining highly appreciable performance, especially for some high-risk patients with chronic kidney disease or contrast agent allergy.

## Study Limitations

Our study had several limitations. First, this was a single center study with a limited number of ACS patients; we need more patients with various complex coronary diseases to further evaluate the safety and efficacy of OCT imaging with VLCCR. Second, the injection rate of each medium may affect image quality except the type of flushing solution. Optimal infusion rate of VLCCR for OCT image acquisition has not been established. Regarding the infusion rate of contrast as recommended injection rate of 4 ml/s for LCA and 3–3.5 ml/s for RCA, the higher rate of 6 ml/s was used to acquire OCT images in both LCA and RCA in the present study due to a lower viscosity compared with contrast. Third, we used different methods to inject solution by manual injection and manual combined with automated injector. Another group using automated injector for contrast will give us more information about images quality comparison between them. Therefore, further investigations are required to utilize the same methods to inject the media and obtain a reasonable rate of medium injection.

## Conclusion

We report here that OCT imaging using VLCCR for blood clearance is feasible and safe and provides similar imaging quality and qualitative measurements compared to OCT imaging using radiographic contrast media for ACS patients.

## Data Availability Statement

The raw data supporting the conclusions of this article will be made available by the authors, without undue reservation.

## Ethics Statement

The studies involving human participants were reviewed and approved by the Institutional Ethics Committee of Harbin Medical University. The patients/participants provided their written informed consent to participate in this study.

## Author Contributions

All authors listed have made a substantial, direct, and intellectual contribution to the work and approved it for publication.

## Funding

This study was supported in part by grants from the National Basic Research Program of China (No. 2016YFC1301103 to BY) and the National Natural Science Foundation of China (No. 81827806 to BY).

## Conflict of Interest

The authors declare that the research was conducted in the absence of any commercial or financial relationships that could be construed as a potential conflict of interest.

## Publisher's Note

All claims expressed in this article are solely those of the authors and do not necessarily represent those of their affiliated organizations, or those of the publisher, the editors and the reviewers. Any product that may be evaluated in this article, or claim that may be made by its manufacturer, is not guaranteed or endorsed by the publisher.
